# L-Arabinose Transport and Metabolism in *Salmonella* Influences Biofilm Formation

**DOI:** 10.3389/fcimb.2021.698146

**Published:** 2021-07-22

**Authors:** Erin M. Vasicek, Lindsey O’Neal, Matthew R. Parsek, James Fitch, Peter White, John S. Gunn

**Affiliations:** ^1^ Center for Microbial Pathogenesis, The Research Institute at Nationwide Children’s Hospital, Columbus, OH, United States; ^2^ Infectious Diseases Institute, The Ohio State University, Columbus, OH, United States; ^3^ Department of Pediatrics, The Ohio State University College of Medicine, Columbus, OH, United States; ^4^ Department of Microbiology, University of Washington, Seattle, WA, United States; ^5^ The Institute for Genomic Medicine, The Research Institute at Nationwide Children’s Hospital, Columbus, OH, United States

**Keywords:** *Salmonella*, biofilm, arabinose, inducible promoters, c-di-GMP

## Abstract

L-arabinose inducible promoters are commonly used in gene expression analysis. However, nutrient source and availability also play a role in biofilm formation; therefore, L-arabinose metabolism could impact biofilm development. In this study we examined the impact of L-arabinose on *Salmonella enterica* serovar Typhimurium (*S*. Typhimurium) biofilm formation. Using mutants impaired for the transport and metabolism of L-arabinose, we showed that L-arabinose metabolism negatively impacts *S.* Typhimurium biofilm formation *in vitro*. When L-arabinose metabolism is abrogated, biofilm formation returned to baseline levels. However, without the ability to import extracellular L-arabinose, biofilm formation significantly increased. Using RNA-Seq we identified several gene families involved in these different phenotypes including curli expression, amino acid synthesis, and L-arabinose metabolism. Several individual candidate genes were tested for their involvement in the L-arabinose-mediated biofilm phenotypes, but most played no significant role. Interestingly, in the presence of L-arabinose the diguanylate cyclase gene *adr*A was downregulated in wild type *S*. Typhimurium. Meanwhile *cyaA*, encoding an adenylate cyclase, was downregulated in an L-arabinose transport mutant. Using an IPTG-inducible plasmid to deplete c-di-GMP *via vieA* expression, we were able to abolish the increased biofilm phenotype seen in the transport mutant. However, the mechanism by which the L-arabinose import mutant forms significantly larger biofilms remains to be determined. Regardless, these data suggest that L-arabinose metabolism influences intracellular c-di-GMP levels and therefore biofilm formation. These findings are important when considering the use of an L-arabinose inducible promoter in biofilm conditions.

## Introduction

*Salmonella enterica* serovar Typhimurium (*S*. Typhimurium) can grow as either individual planktonic cells or as aggregates adhered to a surface known as a biofilm in response to environmental stimuli (Flemming and Wuertz, 2019). These cells are surrounded by a self-produced extracellular matrix (ECM) comprised of proteins, exopolysaccharides, and nucleic acids. While within a biofilm, these bacteria are protected from a variety of challenges and as a result are estimated to be involved in 80% of chronic infections (Davies, 2003). Therefore, further studies are required to identify key aspects of biofilm formation and persistence in order to enhance the efficacy of treating infections associated with biofilms.

The dinucleotide second messenger cyclic-di-GMP (c-di-GMP) has been identified as one of the factors that controls ECM synthesis and adhesion (Caly et al., 2015), regulating the transition between the planktonic and biofilm lifestyles of many bacteria, including *S*. Typhimurium (Solano et al., 2009; Chua et al., 2014; Dahlstrom and O’Toole, 2017). Usually, increased intracellular c-di-GMP promotes biofilm formation, while low levels increase motility and dispersal (Hengge, 2009; Lee et al., 2010; Chua et al., 2015; Valentini and Filloux, 2016; Gao et al., 2017; Liu et al., 2017). In *Salmonella*, the MlrA transcription factor promotes *csgD* transcription whose gene product CsgD stimulates both *adrA* and curli genes (Brown et al., 2001). The diguanylate cyclase, encoded by *adrA*, synthesizes c-di-GMP which then activates cellulose synthase (Zogaj et al., 2001).

Alternatively, the second messenger cyclic adenosine monophosphate (cAMP) also mediates biofilm formation particularly in carbon catabolite repression (Liu et al., 2020). When phosphotransferase system (PTS) sugars are limited and instead non-PTS sugars are present, the adenylyl cyclase gene product of *cyaA* is activated to synthesize cAMP which activates genes encoding the transport of non-PTS sugars into the cell (Park et al., 2006) and represses carbon catabolites (Deutscher et al., 2006). Increased intracellular cAMP also inhibits *mlrA* and *csgD* transcription which reduces biofilm formation in *Salmonella* (Paytubi et al., 2017). This implies that c-di-GMP and cAMP play key roles in the regulation of biofilm formation.

Nutrient availability also plays a role in biofilm formation. While *Salmonella* prefers D-glucose as a carbon source, it can utilize the pentose L-arabinose (Gutnick et al., 1969). Upon transport into the cell *via* the AraE permease (Lee et al., 1981; Lee et al., 1982), L-arabinose is metabolized into substrates for the pentose phosphate pathway (Englesberg, 1961; Englesberg et al., 1962). Intracellular L-arabinose bound to the AraC transcriptional regulator induces expression of the *araBAD* operon and the *araE* gene (Englesberg et al., 1965; Lee et al., 1980; Lee et al., 1981; Lee et al., 1982). In the absence of L-arabinose, AraC acts as a repressor (Schleif, 2010). It is this relationship that is often utilized for conditional, dependent expression of cloned genes, commonly utilized in the plasmid pBAD which contains the promoter of the L-arabinose operon and the regulatory gene *araC* (Guzman et al., 1995).

In this study, while utilizing L-arabinose inducible pBAD strains in *S*. Typhimurium biofilm analysis, we observed a strikingly significant decrease in biofilm formation in the presence of 0.2% L-arabinose. This phenomenon is dependent upon the first step in L-arabinose metabolism, acted upon by the L-arabinose isomerase AraA. However, when *Salmonella* lacks the ability to transport L-arabinose into the cell *via* the high-affinity transport system encoded by *araE*, it instead develops robust biofilms. Despite extensive phenotypic analyses and RNA-Seq expression analysis, the mechanisms by which this occurs remains to be determined. We speculate that under these conditions, the cells respond to carbon starvation by upregulating pathways involved in alternate energy sources.

## Materials And Methods

### Bacterial Strains and Growth Conditions

Wild-type (WT) *Salmonella enterica* serovar Typhimurium ATCC 14028 (JSG210) and its derivatives, were used in these studies **(** **Table S1** **)**. Cultures were first streaked on Luria-Bertani (LB) agar plates and incubated at 37°C overnight. Single colonies were then used to start overnight (O/N) liquid cultures grown in either LB broth, Tryptic Soy Broth (TSB), or Minimal Media (M9) at 37°C on a rotating drum. When grown in the presence of sugar, L-arabinose, D-arabinose, or D-glucose was included at 0.2% or 2% in 1:20 TSB, and 5 mM or 40 mM L-arabinose in M9. Antibiotics, when needed, were used at the following concentrations: ampicillin (Amp), 100 μg/mL or 200 µg/mL; chloramphenicol (Cam), 25 µg/mL; Isopropyl β-D-1-thiogalactopyranoside (IPTG), 2 mM; kanamycin (Kan), 45 µg/mL; and streptomycin (Strep), 100 µg/mL.

### Generation of Mutants and Recombination Procedures

Mutants were created by the λ-red mutagenesis method (Datsenko and Wanner, 2000) with specific primers designed for the gene of interest **(** **Table S2** **)**. Marked gene deletions were transduced into WT using phage P22 HT105/1 *int*-*201* (Maloy et al., 1996). Mutants were verified throughout the process *via* PCR and electrophoretic gel analysis.

### Biofilm Growth and Crystal Violet Assays

*S*. Typhimurium biofilms were grown as follows: Overnight (O/N) cultures were grown in TSB at 37°C with aeration. These were normalized to OD_600_ = 0.8, diluted 1:10 into biofilm growth media (TSB diluted 1:20 or M9 +/- experimental conditions), and 0.1 mL was dispensed in triplicate into non-treated polystyrene 96-well plates (Corning). The plates were incubated for 24 hours (hr) at 25°C on a GyroMini nutating mixer (LabNet International, Inc.) at 24 rpm. If incubation longer than 24 hr was required, media was changed daily.

Attached biofilms of *S*. Typhimurium were then washed twice in ddH_2_O, heat fixed for 1 hr at 60°C, and stained with 0.33% crystal violet for 5 minutes (min). After two subsequent washes in ddH_2_O, the dye was released using 33% acetic acid, and the optical density was measured at 570 nm (OD_570_) in a SpectraMax Spectrophotmeter with SoftMax Pro software (Molecular Devices) to determine the amount of dye retained, which correlates to the amount of biofilm present. All biofilm experiments were performed in triplicate.

### Biofilm CFUs

Biofilm growth was prepared as previously described above, with some modification. After incubation, the planktonic cells were removed and attached biofilms were then washed with 100 μL phosphate-buffered saline (PBS, Gibco-Life Technologies) twice to remove planktonic cells. Biofilms were then scraped for 2 minutes with a 200 µL pipette tip in 100 µL PBS, then diluted 1:10 in 1 mL PBS in microcentrifuge tubes to be vortexed for 3 minutes before being serially diluted 1:10 and 10 µL drip plated onto LB agar and incubated O/N at 37°C for quantification of CFU/mL. Biofilm CFUs were performed in triplicate.

### Growth Curves

*S*. Typhimurium growth curves were performed as follows: O/N cultures were grown in TSB at 37°C with aeration. These were normalized to OD_600_ = 0.8, diluted 1:10 into 4 mL 1:20 TSB, with or without 0.2% or 2% L-arabinose added, in 5 mL screw-cap tubes (VWR). The tubes were incubated for 24 hr at 25°C on a GyroMini nutating mixer (LabNet International, Inc.) at 24 rpm. Aliquots were removed at designated time points, serial diluted 1:10 and 10 µL drip plated onto LB agar and incubated O/N at 37°C for quantification of CFU/mL. Growth curves were done in triplicate as biological replicates and averaged together. Growth curves performed in M9 were also normalized to OD_600_ = 0.8, diluted 1:10 into 1 mL M9 with 5 mM or 40 mM L-arabinose, and 0.1 mL was dispensed in triplicate into non-treated polystyrene 96-well plates (Corning). The plates were incubated for 24 hours (hr) static at 25°C, and the optical density was measured every 30 minutes, shaking for 5 seconds before each read, at 600 nm (OD_600_) in a SpectraMax Spectrophotmeter with SoftMax Pro software (Molecular Devices).

### pH Measurement

The pH of the growth media was measured as follows. Before bacterial inoculation, the pH of 1:20 TSB with or without 0.2% or 2% L-arabinose was measured by a pH Meter (Denver Instrument). O/N cultures were normalized to OD_600_ = 0.8, diluted 1:10 into 20 mL 1:20 TSB, with or without 0.2% or 2% L-arabinose added, in 50 mL screw-cap conical centrifuge tubes (CellPro). The tubes were incubated for 24 hr at 25°C on a GyroMini nutating mixer (LabNet International, Inc.) at 24 rpm until the bacterial culture reached logarithmic growth. Cultures were then pelleted, the supernatant removed to a new 50 mL tube, and the pH was measured.

### Confocal Microscopy of Biofilms

Established biofilms of GFP-expressing strains JSG4093 and JSG4244 were incubated with or without 0.2% L-arabinose for 24 hr prior to imaging. Biofilms were washed two times in PBS and fixed in 2% paraformaldehyde (PFA, Affimetrix) for 1 hour at room temperature and saved for later imaging. The amount of biofilm and the structure of the biofilm can be inferred by the amount of GFP signal detected (Franklin et al., 2015). Biomass and average thickness were assessed by automated capturing of 5 random Z-stacks per well in 4 wells per treatment using a Nikon A1R Live Cell Inverted Confocal microscope. The Z-stacks were then analyzed using the software package COMSTAT2.

### Invasion Assays

HeLa cells (ATCC) were first allowed to attach in surface treated tissue culture flasks (Fisherbrand) Dulbecco’s Modified Eagle Medium (DMEM, Gibco-Life Technologies) + 1% Heat-Inactivated Fetal Bovine Serum (FBS, Corning) and Penicillin-Streptomycin (Gibco-Life Technologies) at 37°C in a humidified incubator with 5% CO_2_ until confluent. After washing with PBS twice to remove any dead cells, the HeLa monolayer was removed with 0.25% Trypsin-EDTA (1X) (Gibco-Life Technologies), cells were washed to remove Trypsin, counted using Trypan Blue (Gibco-Life Technologies), and seeded in DMEM + 1% FBS and strep/pen at a density of approximately 5.0 × 10^5^ cells/well in 24-well polystyrene microplates (Falcon) for infection studies.

Overnight cultures of WT *S*. Typhimurium grown in LB were backdiluted 1:100 and grown statically in LB +/- 0.2% L-arabinose for 3 hr at 37°C. Bacteria were equilibrated in DMEM to an OD_600_ of 0.8. HeLa cells were washed with PBS twice to remove any dead cells and bacteria were added at a multiplicity of infection (MOI) of 100 in 1 mL DMEM + 1% FBS. Extracellular bacteria were removed 1 hr post-infection by addition of 50 μg/ml of gentamicin (Gibco-Life Technologies) for 1 hr followed by washing with PBS three times to remove additional extracellular bacteria. The infected HeLa cells were lysed with 0.1% Triton X-100 (Calbiochem) for 15 min. The cell lysates were then serially diluted, plated onto LB agar and enumerated after 24 hr incubation at 37°C. Invasion assay was performed in triplicate.

### RNA Isolation

Biofilm growth was as described above with some modification. Each strain was grown in an entire 96-well plate for four days, with media replenished daily. After incubation, the biofilm was washed with 100 μL phosphate-buffered saline (PBS) twice to remove planktonic cells, scraped for 2 minutes with a 200 μL pipette tip, transferred to a microcentrifuge tube, and centrifuged at 10,000 × *g* for 3 min. RNA was extracted using the hot phenol method. Briefly, the supernatant was removed, and pellets were resuspended in 475 μl AE buffer (50 mM sodium acetate [NaOAc], 10 mM EDTA, pH 5.2). Forty microliters of 20% SDS and 475 μl of phenol were added and incubated for 10 min at 65°C, shaking every minute. Samples were put on ice for 5 min and centrifuged for 15 min at 10,000 rpm at 4°C. Supernatants were transferred to new microcentrifuge tubes and 475 μL of chloroform was added, mixed, and centrifuged for 10 min at 2,000 rpm at 4°C. The top aqueous layer was placed in a new microcentrifuge tube, and RNA was precipitated by adding 500 μL isopropanol and 50 μL sodium acetate (2 M), centrifuging for 20 min (12,000 rpm, 4°C), and washing with 250 μL 70% cold ethanol. Finally, samples were treated with DNase (12.5% of total reaction volume, catalog number M0303L; New England BioLabs, Ipswich, MA, USA) for 45 min. RNA isolation was performed in quadruplicate.

### RNA-Seq Library Construction and Sequencing

Total RNA quality was assessed using an Agilent 2100 bioanalyzer and RNA Nano Chip kit (Agilent Technologies) to ensure that the RNA integrity number (RIN) was ≥7 and that there were no RIN value outliers. rRNA was removed from 1 μg of RNA with a Ribo-Zero rRNA removal kit for bacteria (Epicentre Biotechnologies). To generate a directional signal in the RNA-Seq data, libraries were constructed from first-strand cDNA using the ScriptSeq v2 RNA-Seq library preparation kit (Epicentre Biotechnologies). Briefly, 50 ng of rRNA-depleted RNA was fragmented and reverse transcribed using random primers containing a 5ʹ tagging sequence, followed by 3ʹ-end tagging with a terminus-tagging oligonucleotide to yield di-tagged single-stranded cDNA. Following purification by a magnetic bead-based approach, the di-tagged cDNA was amplified by limit-cycle PCR using primer pairs that anneal to tagging sequences and add adaptor sequences required for sequencing cluster generation. Amplified RNA-Seq libraries were purified using an AMPure XP System (Beckman Coulter). The quality of libraries was determined *via* an Agilent 2200 TapeStation using high sensitivity D1000 tape and quantified using a Kapa SYBR Fast qPCR kit (KAPA Biosystems, Inc). One hundred fifty-base pair sequence reads were generated using the Illumina HiSeq 4000 platform.

### RNA-Seq Data Analysis

Each sample was aligned to the GCF_000022165.1 assembly of the *S.* Typhimurium strain 14028S reference from NCBI (NC_016856.1) using version 0.7.5a of the BWAMEM aligner (Li and Durbin, 2010). Features were identified from the GFF file that came with the assembly from NCBI. Feature coverage counts were calculated using HTSeq (Anders et al., 2015). Differentially expressed features were calculated using DESeq2 (Love et al., 2014) and custom scripts developed in-house to perform RNA-Seq analysis. The data discussed in this publication have been deposited in NCBI’s Gene Expression Omnibus and are accessible through GEO Series accession number GSE171196 (https://www.ncbi.nlm.nih.gov/geo/query/acc.cgi?acc=GSE171196).

### Statistical Analysis

Statistical analyses were performed using GraphPad Prism software. All analyses were performed on independent experiments done in triplicate, unless otherwise indicated in the methods above, and mean ± the standard deviation was determined. The specific statistical test used are noted in each figure legend.

For RNA-Seq analysis, significant differentially expressed features between the two groups are those that have an absolute value of fold change of ≥1.5 and an adjusted *P* value of ≤0.10 (10% false discovery rate [FDR]).

### Mass Spectometry

C-di-GMP was extracted from *S.* Typhimurium grown in 96-well plates using a method adapted from (Hickman and Harwood, 2008; Irie et al., 2012). Briefly, all experiments were performed on independent cultures in biological triplicates. C-di-GMP was extracted by addition of 9 µl of 70% perchloric acid to each well of the 96 well plate and incubation on ice for 1 hour. After an hour, attached cells were dislodged by pipetting and the supernatant was collected and neutralized using potassium bicarbonate. 2-chloro AMP was used as an internal standard. Liquid chromatography MS/MS measurements were performed using an Acuity UPLC with a Synergi 4μ Hydro RP 80A column and a C18 Guard Cartridge (Phenomenex) on a Premier XL triple-quadrupole electrospray mass spectrometer (Waters). The m/z 691 > 152 transition was used for c-di-GMP and 382 > 170 for 2-chloro-AMP. The cone voltages and collision energies were 40 V/30 eV and 35 V/20 eV, respectively. Thirty microliters of each sample was injected and the ratio of area under the curve of the c-di-GMP channel signal (retention time= 1.6 minutes) was divided by the area under the curve of the 2-chloroAMP signal (retention time= 2.1 minutes). A standard curve of 0 nM to 100 nM c-di-GMP containing 2-chloroAMP was used to quantify c-di-GMP for all samples. C-di-GMP concentration was normalized to total protein concentration.

## Results

### L-Arabinose Metabolism and Import Impact Biofilm Development

**Figure 1:**
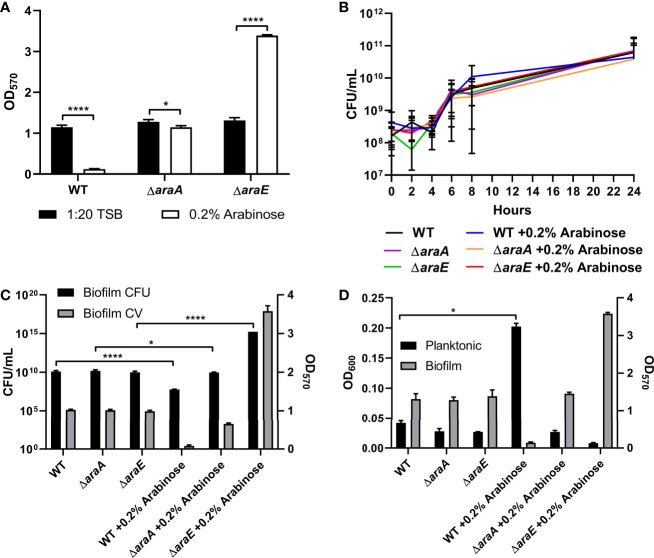
L-arabinose affects biofilm formation but not growth. **(A)** WT 14028, Δ*araA* and Δ*araE* strains were grown in 96-well plates in 100 µL 1:20 TSB (black bars) or 1:20 TSB with 0.2% L-arabinose (white bars). After 24 hours, planktonic cells were removed, then biofilms were washed, heat fixed, and stained with crystal violet (CV) for relative biofilm measurement as determined at OD_570_. **(B)** WT 14028, Δ*araA* and Δ*araE* strains were grown in 5 mL tubes in 4 mL 1:20 TSB or 1:20 TSB with 0.2% L-arabinose. Aliquots were removed at designated time points and plated for colony forming units (CFU). **(C)** After 24 hours, biofilms were removed for CFU enumeration compared to CV staining (OD_570_). **(D)** After 24 hours, planktonic cell density was determined at OD_600_ compared to biofilm CV staining (OD_570_). Data are mean ± SD, statistical analyses were done using a two-way ANOVA with Dunnett’s multiple comparisons test *P < 0.05; ****P < 0.0001.

While utilizing *S*. Typhimurium strains containing the L-arabinose inducible pBAD plasmid controlling genes of interest grown in a biofilm with or without the addition of 0.2% L-arabinose, we observed a dramatic decrease in biofilm formation in our control. Similarly, wild-type *S*. Typhimurium (called WT) biofilm formation was significantly impaired in the presence of L-arabinose (**Figure 1A**). In an effort to determine if this phenotype was due to metabolism or import, we deleted the genes *araA* (encoding L-arabinose isomerase, first step in metabolism) and *araE* (L-arabinose/proton symport protein) in the WT background (**Figure 1A**). Without the presence of L-arabinose, the mutant strains showed no marked change in their biofilm forming abilities from WT. However, when grown in the presence of L-arabinose, without the ability to metabolism L-arabinose *via* AraA, *S*. Typhimurium regained biofilm growth similar to without L-arabinose, suggesting that the metabolism of L-arabinose is involved in the reduction in biofilm formation. Interestingly, when grown in the presence of L-arabinose, losing the ability to import L-arabinose *via* AraE resulted in a dramatic increase in biofilm formation. These data suggested that while metabolism of L-arabinose *via* AraA resulted in decreased biofilm formation, the inability to import L-arabinose *via* AraE increased biofilm formation.

**Figure 2:**
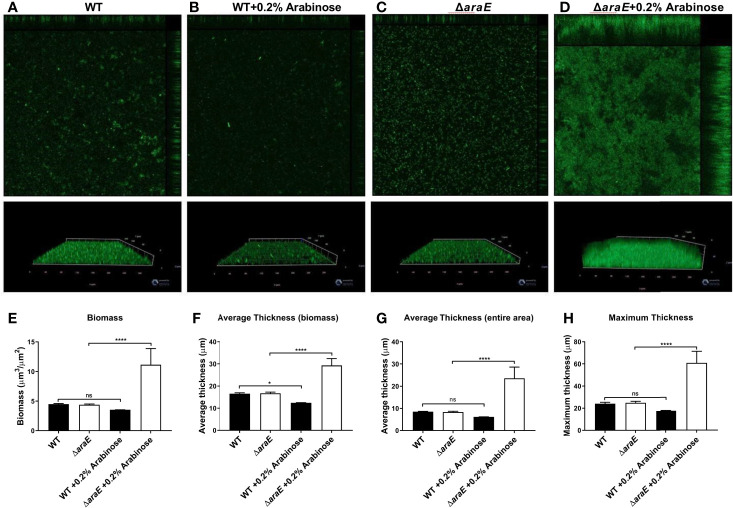
L-arabinose biofilms visualized by confocal microscopy. WT 14028 **(A, B)** and Δ*araE* **(C, D)** GFP-expressing strains were grown in 96-well plates in 100 µL 1:20 TSB (black bars) or 1:20 TSB with 0.2% L-arabinose (white bars). After 24 hours, planktonic cells were removed, then biofilms were washed, fixed in 2% paraformaldehyde, and biofilm structure was determined by GFP signal detection. Five random Z-stacks were captured per well and analyzed for biomass **(E)**, average thickness **(F, G)**, and maximum thickness **(H)** by COMSTAT2. Data are mean ± SD, statistical analyses were done using a two-way ANOVA with Dunnett’s multiple comparisons test NS, not significant (*P* > 0.05); *P < 0.05; ****P < 0.0001.

The characteristics of these biofilms were then examined. First, growth curves demonstrated that the biofilm phenotypes were not attributed to any planktonic growth defect (**Figure 1B**). Next, colony forming units (CFUs) were enumerated from each biofilm. As expected, when the biofilms are reduced in size, as determined by crystal violet (CV) staining, there is a correlation to reduced CFUs. When grown in the presence of L-arabinose, the smaller WT biofilm contained less cells while the larger Δ*araE* biofilm had significantly more (**Figure 1C**). This also correlated to an inverse relationship in a single well where more bacteria existed in a planktonic state when less existed in a biofilm state, and vice versa (**Figure 1D**). Using GFP-tagged strains, we also visualized the biofilm structure in the presence or absence of 0.2% L-arabinose by confocal microscopy (**Figure 2**). In the presence of L-arabinose, the diminished WT biofilm appears as a flat, immature biofilm (**Figure 2B**) while the large Δ*araE* biofilm is clumpy and thick (**Figure 2D**). In agreement with these observations, the Δ*araE* biofilm grown in the presence of L-arabinose was significantly larger when random Z-stacks were measured for average thickness, biomass, and maximum thickness (**Figures 2E** **–H**). All of these characteristics align with the observed phenotypes.

### High L-Arabinose Concentrations Eliminate the *araE* Mutant Hyperbiofilm Phenotype

**Figure 3:**
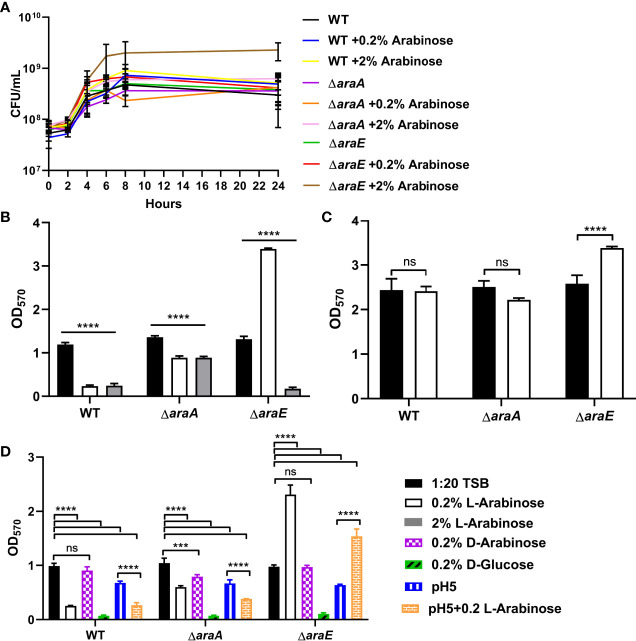
L-arabinose effects biofilm formation at low or high concentrations but does not induce dispersal. **(A)** WT 14028, Δ*araA* and Δ*araE* strains were grown in 5 mL tubes in 4 mL 1:20 TSB with or without 0.2% or 2% L-arabinose. Aliquots were removed at designated time points and plated for colony forming units (CFU). **(B)** WT 14028, Δ*araA* and Δ*araE* strains were grown in 96-well plates in 100 µL 1:20 TSB (black bars) or 1:20 TSB with 0.2% (white bars) or 2% L-arabinose (gray bars). After 24 hours, planktonic cells were removed, then biofilms were washed, heat fixed, and stained with crystal violet (CV) for relative biofilm measurement as determined at OD_570_. **(C)** WT 14028, Δ*araA* and Δ*araE* strains were grown in 96-well plates in 100 µL 1:20 TSB. After 24 hours media was replenished with more 1:20 TSB (black bars) or 1:20 TSB with 0.2% L-arabinose and allowed to grow another 24 hours before staining with CV. **(D)** WT 14028, Δ*araA* and Δ*araE* strains were grown in 96-well plates in 100 µL 1:20 TSB (black bars), 1:20 TSB with 0.2% (white bars), 0.2% D-arabinose (purple bars), 0.2% D-glucose (green bars), pH5 (blue bars), or pH 5 with 0.2% L-arabinose (orange bars). Data are mean ± SD, statistical analyses were done using a two-way ANOVA with Dunnett’s multiple comparisons test NS, not significant (*P* > 0.05); ***P < 0.001; ****P < 0.0001.

We examined the effects of different concentrations of L-arabinose on biofilm formation. In *Escherichia coli* (*E. coli*), there are two L-arabinose import systems: high-affinity (AraE) and low-affinity (AraFHG). However, only the high-affinity system has been identified in *S*. Typhimurium. At low concentrations, such as 0.2%, L-arabinose is transported through AraE in *E. coli* (Lee et al., 1981; Stoner and Schleif, 1983) and *S*. Typhimurium (Lee et al., 1981; Lee et al., 1982). At high concentrations, such as 2%, L-arabinose is also transported through AraFHG in *E. coli* (Hogg and Englesberg, 1969; Schleif, 1969; Brown and Hogg, 1972; Horazdovsky and Hogg, 1989; Luo et al., 2014). If the low-affinity transport system is conserved in *S*. Typhimurium, then a higher concentration of L-arabinose may circumvent the high-affinity transport defect in an Δ*araE* mutant. No growth differences were observed between 1:20 TSB and 2% L-arabinose for WT or Δ*araA*, but Δ*araE* grew slightly better in the presence of 2% L-arabinose (**Figure 3A**). Interestingly, when grown in the presence of 2% L-arabinose the large biofilm phenotype of Δ*araE* observed in the presence of 0.2% L-arabinose was abolished and resembled that of WT grown in the presence of 0.2% L-arabinose (**Figure 3B**). This implies that at higher concentrations, L-arabinose may be imported into the cell by an AraE-independent, low-affinity import system yet to be identified. We tested two candidates, AraJ and MglC, but upon deletion neither impacted import (**Supplemental Figure S1**). These results further suggest that the inability to transport L-arabinose into the cell plays a role in forming larger biofilms. Meanwhile, Δ*araA* retained its ability to form biofilms no matter the concentration of L-arabinose, in agreement with the predicted role of L-arabinose metabolism in decreasing biofilm formation.

### L-Arabinose Does Not Disperse Biofilms

Next, we sought to determine if L-arabinose is not only involved in biofilm formation but if it could also disrupt a preformed biofilm. After 24 hours of biofilm growth without L-arabinose, 0.2% L-arabinose was added to the growth media and biofilms were allowed to grow for another 24 hours prior to CV staining. WT and Δ*araA* biofilms were not affected by the addition of L-arabinose, but in the presence of L-arabinose, Δ*araE* formed more biofilm than without L-arabinose (**Figure 3C**). This suggested that L-arabinose impacted the ability of the biofilm to form but cannot disrupt preexisting biofilm structures.

### Other Pentoses Affect Biofilm Formation

The preferred carbon source for *S*. Typhimurium is D-glucose, but it does possess the ability to utilize other sugars as carbon sources (Kenyon et al., 2005). Therefore, we tested if the same phenomenon observed with L-arabinose in biofilm formation could also be observed with the D-arabinose stereoisoform or D-glucose. The addition of 0.2% D-arabinose to the biofilm growth media had no effect on biofilm formation for WT, Δ*araA*, or Δ*araE*. This agrees with previous studies showing that *S*. Typhimurium cannot metabolize D-arabinose as the sole carbon source (Gutnick et al., 1969), except in certain mutants (Old and Mortlock, 1977). Interestingly, the addition of D-glucose diminished biofilm formation of WT, Δ*araA* and Δ*araE* (**Figure 3D**). Therefore, the phenotype we observed is primarily specific to the L-arabinose, non-phosphotransferase system of uptake and not shared with other pentoses tested.

### Acidification May Play a Role in Biofilm Formation

Previous studies have suggested that acidification of the media occurs as a result of L-arabinose metabolism (López-Garrido et al., 2015). Indeed, we also observed a reduction of pH when WT was grown in the presence of both 0.2% and 2% L-arabinose, Δ*araA* showed no change in pH (likely due to the inability to metabolize L-arabinose), and acidification only occurred when grown in the presence of 2% L-arabinose for Δ*araE* (**Table 1**). As such, we also examined biofilm formation in pH5 with or without L-arabinose. While we did observe slight reductions in biofilm formation in acidic conditions overall, the trends of biofilm formation for WT, Δ*araA*, and Δ*araE* mutant strains remained the same in the presence or absence of L-arabinose in pH5 (**Figure 3C**). This would suggest that while a lower pH does reduce biofilm growth, is not the sole factor contributing to the L-arabinose dependent changes in biofilm formation we observed.

**Table 1:** pH of the culture medium measured before bacterial inoculation (initial) and after the bacterial culture reached logarithmic growth for WT, Δ*araA*, and Δ*araE* in 1:20 TSB, +0.2% L-arabinose, and +2% L-arabinose.

	Initial	WT	Δ*araA*	Δ*araE*
**1:20 TSB**	6.83	6.14	6.14	6.19
**+0.2% L-arabinose**	6.81	4.69	6.14	6.06
**+2% L-arabinose**	6.92	4.20	6.09	4.53

### Hyperbiofilm Formation in Δ*araE* Requires the Extracellular Matrix

**Figure 4:**
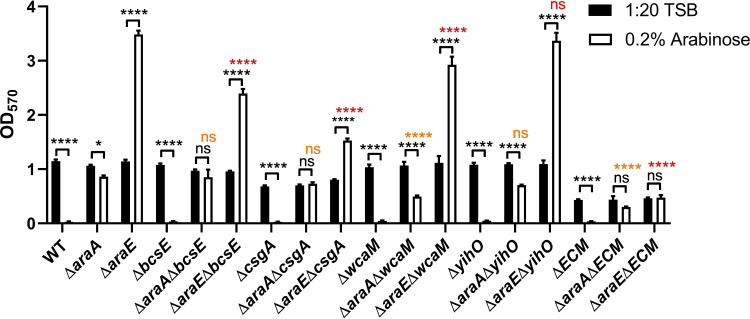
Biofilm formation in the presence of L-arabinose partly depends on the ECM. WT 14028, Δ*araA*, Δ*araE*, and various combinations of ECM mutant strains were grown in 96-well plates in 100 µL 1:20 TSB (black bars) or 1:20 TSB with 0.2% L-arabinose (white bars). After 24 hours, planktonic cells were removed, then biofilms were washed, heat fixed, and stained with crystal violet (CV) for relative biofilm measurement as determined at OD_570_. Data are mean ± SD, statistical analyses were done using a two-way ANOVA with Dunnett’s multiple comparisons test NS, not significant (*P* > 0.05); *P < 0.05; ****P < 0.0001. Orange text compares Δ*araA* in 0.2% L-arabinose to its respective double mutant in 0.2% L-arabinose. Red text compares Δ*araE* in 0.2% L-arabinose to its respective double mutants in 0.2% L-arabinose.

Since the effect of L-arabinose on biofilm formation likely occurs early in attachment or development, we tested mutants in four major components of the extracellular matrix (ECM): cellulose (*bcsE*), curli (*csgA*), O-antigen (*yihO*), and colonic acid (*wcaM*) (Prouty et al., 2002; Solano et al., 2002; Ledeboer and Jones, 2005; Gibson et al., 2006; Jonas et al., 2007; Gunn et al., 2016). Single mutants and a combination of all four components in the ΔECM mutant were subjected to biofilm growth assays with or without 0.2% L-arabinose. Double mutants were also created pairing each mutant with either Δ*araA* or Δ*araE* (**Figure 4**). The Δ*csgA*, Δ*bcsE*, and ΔECM mutants have been previously shown to have impaired biofilms (Adcox et al., 2016). Even though both the ΔECM and Δ*csgA* mutants form poor biofilms under normal conditions, the addition of L-arabinose does further inhibit the ability to form a biofilm. As previously observed in the WT background, deletion of *araA* from each single mutant and the ΔECM strain in the presence of L-arabinose resulted in increased biofilm development (often to similar levels as the single mutant alone in 1:20 TSB) when compared to each single mutant alone in the presence of L-arabinose. Deletion of *araE* in each of the single mutant backgrounds resulted in significant increases in biofilm development in the presence of L-arabinose, but in the Δ*csgA*, Δ*bcsE*, and Δ*wcaM* mutant backgrounds, the magnitude of the increase was significantly less (noted in red). Although statistically different, the biofilm formation of Δ*araE*Δ*wcaM* in the presence of L-arabinose was still quite robust and we did not consider its reduction biologically important. However, combined deletion of all four ECM components abolished the hyperbiofilm phenotype of Δ*araE* in the presence of L-arabinose. This suggests there is an interplay among the ECM components that is required for the hyperbiofilm phenotype of the Δ*araE* strain when grown with L-arabinose.

### L-Arabinose Affects Host Cell Invasion

**Figure 5:**
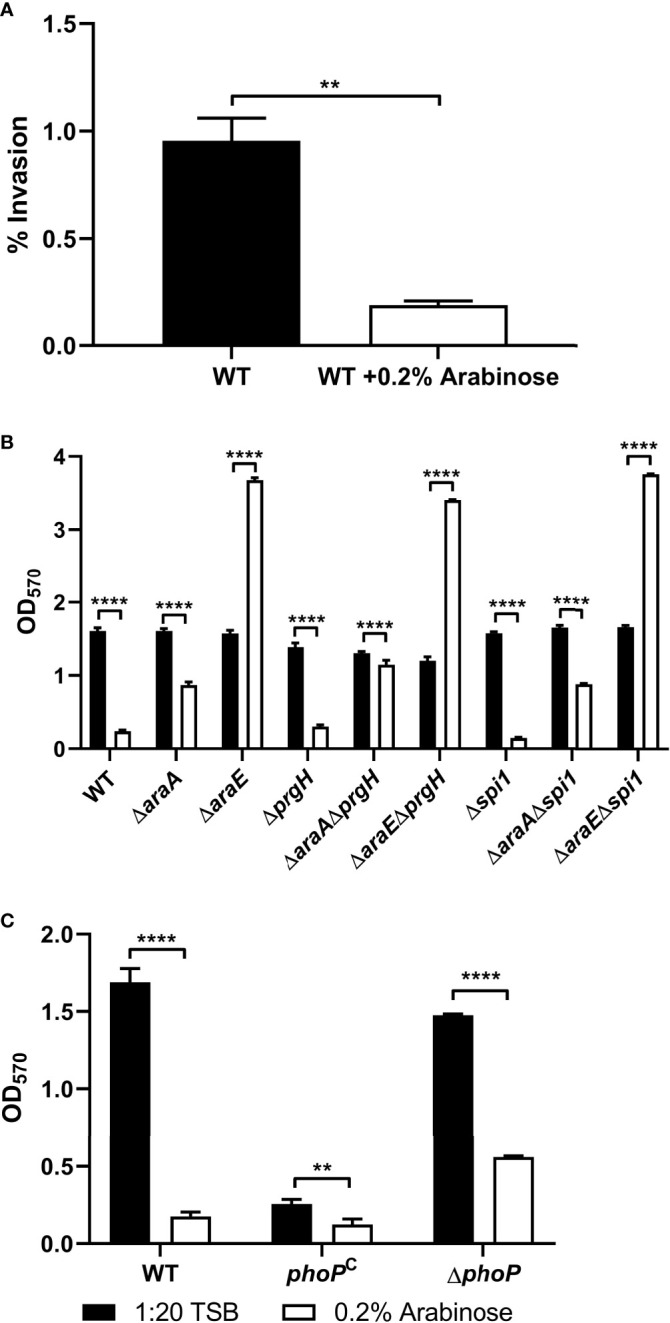
L-arabinose effects host cell invasiveness but invasion genes do not affect biofilm formation. **(A)** HeLa monolayer cells were infected with WT 14028 cells at an MOI of 100 for 1 hour before being washed with 1xPBS and treated with 50 µg/mL gentamicin to remove any extracellular bacteria. Cell lysates were plated for CFU enumeration. WT 14028, Δ*araA*, Δ*araE*, and various combinations of SPI-1 mutant strains **(B)** or *phoP* strains **(C)** were grown in 96-well plates in 100 µL 1:20 TSB (black bars) or 1:20 TSB with 0.2% L-arabinose (white bars). After 24 hours, planktonic cells were removed, then biofilms were washed, heat fixed, and stained with crystal violet (CV) for relative biofilm measurement as determined at OD_570_. Data are mean ± SD, statistical analyses were done using **(A)** Welch’s unpaired t-test or **(B, C)** two-way ANOVA with Dunnett’s multiple comparisons test NS, not significant (*P* > 0.05); **P < 0.01; ****P < 0.0001.

Previous studies have shown that *Salmonella* Pathogenicity Island 1 (SPI-1) is repressed by L-arabinose *via* posttranscriptional interactions with HilD, the transcriptional regulator of SPI-1, thus inhibiting invasion of HeLa cells (López-Garrido et al., 2015). In those studies, neither AraC nor AraA played a major role, and instead the import of L-arabinose *via* AraE was required for repression. Using HeLa cells grown in a monolayer infected with a multiplicity of infection (MOI) of 100, we confirmed that L-arabinose does impact invasion of HeLa cells (**Figure 5A**). This led us to inquire if this regulation of SPI-1 could also impact biofilm formation. We tested a Δ*prgH* mutant (type III secretion system protein encoded on SPI-1) and a Δ*spi1* mutant lacking the entire SPI-1 operon alone, or combined with deletions in *araA* and *araE*, for their ability to form biofilms in the presence or absence of L-arabinose. There was no significant alteration of biofilm formation of any of the strains tested (**Figure 5B**). This suggests that the change in SPI-1 expression regulated by L-arabinose does not impact biofilm formation.

Another regulator of virulence and biofilm formation is the two-component regulatory system PhoP/PhoQ (Fields et al., 1989; Miller et al., 1989; Prouty and Gunn, 2003). To test whether this regulatory system plays a role in L-arabinose mediated biofilm alteration, we tested a PhoP deletion mutant and a PhoP constitutively active strain (*phoP* ^C^) in the presence and absence of L-arabinose (**Figure 5C**). The *phoP* ^C^ strain alone exhibits poor biofilm formation but like WT, was further reduced in the presence of L-arabinose. Meanwhile, Δ*phoP* forms biofilms similar to WT, but these biofilms were still reduced in the presence of L-arabinose. Therefore, the PhoP/PhoQ regulators, while affecting biofilm formation, were not involved in L-arabinose mediated biofilm modulation.

### RNA-Seq Reveals Genes Affected by L-Arabinose

**Figure 6:**
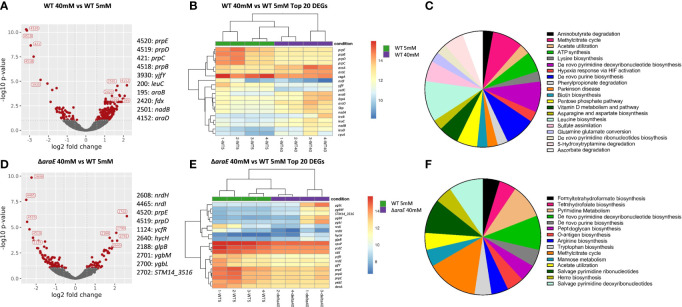
Differentially expressed genes in biofilms grown in the presence of L-arabinose. For RNA-Seq analysis, biofilms were grown for 4 days in M9 with 5 mM and 40 mM L-arabinose. **(A, D)** Volcano plots for WT 40 mM versus WT 5 mM and Δ*araE* 40 mM and WT 5 mM L-arabinose. The *x-*axis specifies the fold change (FC) and the *y-*axis specifies the negative logarithm to the base 10 of the *t* test *P* values. Dashed lines represent the filtering criteria (FC ≥ 1.5, *P* ≤ 0.05). Red dots represent probe sets for transcripts expressed at significantly higher or lower levels. The top 5 up and down regulated genes are labeled. **(B, E)** Dendrogram and unsupervised hierarchical clustering heat map (using Euclidean distance) of gene expression based on the log ratio fragments per kilobase per million mapped reads (FPKM) data. Each column represents a biological replicate from each experimental condition; rows represent genes. Red, upregulation; blue, downregulation. The vertical distances on each branch of the dendrogram represent the degrees of similarity between gene expression profiles of various groups. **(C, F)** Pathways of enriched genes in each experimental condition as determined by Panther.

To better identify pathways involved in the response to L-arabinose we performed an RNA-Seq analysis comparing biofilms with or without L-arabinose (**Figures 6A–F**). For these studies, we used M9 minimal media because it is a defined medium for which we could control the exact amount of sugar present, in this case L-arabinose as the sole carbon source. We first determined concentrations of L-arabinose that phenocopied the biofilm results of WT *S*. Typhimurium in 1:20 TSB with 0.2% L-arabinose. These concentrations were 5 mM L-arabinose (mimicking 1:20 TSB alone) and 40 mM L-arabinose (producing the same altered biofilm formation seen in 1:20 TSB with 0.2% L-arabinose) (**Supplemental Figure S2A**). The Δ*araA* mutant was excluded from RNA-Seq analysis because it was unable to grow at any concentration of L-arabinose. The Δ*araE* mutant was able to grow in M9 supplemented with L-arabinose, further suggesting that L-arabinose was able to be internalized *via* a non-AraE mechanism. Also, at higher concentrations of L-arabinose, the increased biofilm formation of the Δ*araE* mutant was abolished as observed in 1:20 TSB with 2% L-arabinose. There was no observed growth defect for WT or Δ*araE* when grown in M9 supplemented with 40 mM L-arabinose (**Supplemental Figure S2B**).

As expected, the entire L-arabinose operon was upregulated when WT was grown in low (5 mM) versus high (40 mM) L-arabinose: *araA* 27.8-fold, *araB* 23.5-fold, *araC* 2.8-fold, *araD* 10.3-fold, *araE* 10.3-fold, *ygeA* 7.0-fold (putative aspartate racemase 50% cotranscribed with *araE*), and *araU*/STM14_0177 2.4-fold (Stringer et al., 2014). Several of these genes were among the most differentially regulated genes (**Table 2**). Additionally, the curli fimbriae genes were highly downregulated: *csgA* -30.2-fold, *csgB* -41.4-fold, *csgC* -3.6-fold. To better understand how L-arabinose affects gene expression, the Panther classification system tool was used to perform a functional analysis (Mi et al., 2013), identifying relevant biological processes. The majority of the differentially expressed genes were unclassified, but identified pathways included genes involved in the pentose phosphate pathway, TCA cycle, amino acid biosynthesis, pyrimidine and purine biosynthesis, and ATP synthesis (**Figure 6C** and **Supplemental Table S3**).

**Table 2:** Top differentially regulated genes in WT biofilms grown in 40 mM L-Arabinose compared to 5 mM by RNA-Seq^a^.

Gene	Fold change	Function
***araA***	**27.8**	**L-arabinose isomerase**
***araB***	**23.51**	**ribulokinase**
*ibpA*	18.67	heat shock protein IbpA
***araE***	**15.23**	**L-arabinose/proton symport protein**
***araD***	**10.34**	**L-ribulose-5-phosphate 4-epimerase**
*groES*	10.23	co-chaperonin GroES
*putA*	7.31	Bifunctional protein PutA
*ygeA*	7.04	putative racemase
*groEL*	6.8	chaperonin GroEL
*sbp*	6.69	sulfate transporter subunit
*cysA*	6.49	sulfate/thiosulfate transporter subunit
*cysP*	6.09	thiosulfate transporter subunit
*treB*	5.82	pseudogene
*cysD*	5.79	sulfate adenylyltransferase subunit 2
*htpG*	5.63	heat shock protein 90
*leuD*	5.29	3-isopropylmalate dehydratase small subunit 1
*ybbN*	5.13	putative thioredoxin protein
*STM14_4895*	5.04	putative mannose-6-phosphate isomerase
*yneC*	5.04	autoinducer-2 (AI-2) modifying protein LsrG
*putP*	5.03	major sodium/proline symporter
*leuC*	4.97	isopropylmalate isomerase large subunit
*nadA*	4.93	quinolinate synthetase
*yneA*	4.76	putative sugar transport protein
*fadL*	4.73	long-chain fatty acid outer membrane transporter
*cysI*	4.71	sulfite reductase subunit beta
*nmpC*	4.7	putative outer membrane porin precursor
*yneB*	4.57	aldolase
*cysW*	4.5	Thiosulfate permease W protein
*STM14_1513*	4.47	putative outer membrane lipoprotein
*asnB*	4.27	Asparagine synthetase B
*zraP*	4.05	Zinc resistance-associated protein
*fdx*	3.93	electron carrier protein
*nadB*	3.9	L-aspartate oxidase
*csgC*	-3.56	putative autoagglutination protein
*nrdE*	-4.09	ribonucleotide-diphosphate reductase subunit alpha
*adrA*	-4.25	diguanylate cyclase AdrA
*sitC*	-4.94	putative permease
*sitB*	-5.22	putative ATP-binding protein
*ycdC*	-5.25	putative transcriptional repressor
*STM14_1829*	-5.37	putative cytoplasmic protein
*sitD*	-5.46	putative permease
*nrdI*	-5.47	Protein NrdI
*ymdF*	-16.91	putative cytoplasmic protein
*yjfY*	-21.02	putative outer membrane protein
*csgA*	-30.21	cryptic curlin major subunit
*csgB*	-41.36	Minor curlin subunit
*prpE*	-44.53	Propionate–CoA ligase
*prpD*	-45.07	2-methylcitrate dehydratase
*prpC*	-45.15	methylcitrate synthase
*prpB*	-46.08	Serine/threonine-protein phosphatase 2

a Arabinose genes are in boldface font.

In the Δ*araE* biofilms formed in the presence of high (40 mM) L-arabinose, none of the L-arabinose metabolism genes were upregulated, implying that although this sugar can enter the cell through an AraE-independent mechanism, it was not enough to activate the L-arabinose pathway. Instead the most highly upregulated genes were involved in threonate catabolism while the most downregulated were uridine phosphorylase and cytidine deaminase (**Table 3**). Surprisingly, curli genes *csgA* (-6.0-fold) and *csgC* (-2.6-fold) were downregulated in Δ*araE* biofilms contrary to the observed robust biofilms in this mutant in the presence of L-arabinose. Other pathways identified by the Panther classification system tool which experienced differential gene expression changes included those involved in formate biosynthesis, TCA, cycle, amino acid biosynthesis and metabolism, pyrimidine biosynthesis, metabolism, and salvage, purine biosynthesis, and ATP synthesis (**Figure 6F** and **Supplemental Table S3**).

**Table 3:** Top differentially regulated genes in Δ*araE* biofilms grown in 40 mM L-Arabinose compared to WT 5 mM L-Arabinose by RNA-Seq.

Gene	Fold Change	Function
*ygbK*	49.07	putative tRNA synthase
*ygbL*	33.96	putative aldolase
*ygbJ*	31.76	3-hydroxyisobutyrate dehydrogenase
*ygbM*	17.59	hypothetical protein
*STM14_3516*	7.09	putative nucleoside-diphosphate-sugar epimerase
*glpF*	6.2	glycerol diffusion
*glpK*	5.87	Glycerol kinase
*hycH*	5.38	hydrogenase 3 large subunit processing protein
*hycD*	5.15	Hydrogenase 3, membrane subunit
*STM14_3515*	5.03	putative permease
*hycG*	4.82	hydrogenase
*argH*	4.75	argininosuccinate lyase
*hycI*	4.46	Protease involved in processing C-terminal end of HycE
*hycF*	4.43	formate hydrogenlyase complex iron-sulfur subunit
*glpB*	4.27	anaerobic glycerol-3-phosphate dehydrogenase subunit B
*glpT*	3.98	MFS family, sn-glycerol-3-phosphate transport protein
*STM14_2886*	3.53	putative transketolase
*ulaA_2*	3.48	ascorbate-specific PTS system enzyme IIC
*STM14_3071*	-3.51	putative inner membrane protein
*ygaP*	-3.52	putative rhodanese-like sulfurtransferase
*maf_2*	-3.53	Maf-like protein
*folA*	-3.61	Dihydrofolate reductase
*otsB*	-3.7	trehalose-6-phosphate phosphatase
*yejG*	-3.86	hypothetical protein
*yqjI*	-3.87	putative transcriptional regulator
*lexA*	-4.02	LexA repressor
*yebG*	-4.05	DNA damage-inducible protein YebG
*STM14_2428*	-4.16	hypothetical protein
*deaD*	-4.59	ATP-dependent RNA helicase DeaD
*nrdE*	-4.65	ribonucleotide-diphosphate reductase subunit alpha
*STM14_3374*	-4.95	putative regulatory protein
*nrdH*	-5.83	glutaredoxin-like protein
*csgA*	-6.01	cryptic curlin major subunit
*sulA*	-6.52	SOS cell division inhibitor
*deoC*	-6.56	deoxyribose-phosphate aldolase
*prpB*	-6.77	Serine/threonine-protein phosphatase 2
*yjfY*	-7.36	putative outer membrane protein
*nrdI*	-7.49	Protein NrdI
*rpoH*	-8.44	RNA polymerase factor sigma-32
*prpC*	-8.59	methylcitrate synthase
*yebE*	-8.86	putative inner membrane protein
*deoA*	-9.35	thymidine phosphorylase
*prpD*	-12.63	2-methylcitrate dehydratase
*cspA*	-12.86	major cold shock protein
*cpxP*	-14.03	Periplasmic repressor of cpx regulon by interaction with CpxA
*ycfR*	-14.23	putative outer membrane protein
*prpE*	-14.98	Propionate–CoA ligase
*udp*	-31.76	uridine phosphorylase
*cdd*	-40.17	cytidine deaminase
*ycdZ*	-71.89	putative inner membrane protein

### The *ycfR* and *cyaA* Genes Are Not Responsible for the Arabinose-Mediated Δ*araE* Robust Biofilm Phenotype

Of particular interest, within the differentially expression genes observed by RNA-Seq in the Δ*araE* mutant grown with 40 mM L-arabinose was the downregulation of *ycfR* (-14.2-fold). This gene encodes a putative outer membrane protein previously identified to be responsive to L-arabinose in planktonic growth conditions and when deleted forms large biofilms that resemble those seen in this study (Gonzalez-Escobedo and Gunn, 2013). To test whether constitutive expression of *ycfR* could reverse our observed biofilm phenotype, we created a plasmid containing *ycfR* under control of the *lacZ* promoter and introduced it into an Δ*araE* mutant strain. However, overexpression of *ycfR* was unable to alter biofilm formation of Δ*araE* in the presence of L-arabinose (**Supplemental Figure S3A**). Therefore, downregulation of *ycfR* does not play a role in the hyperbiofilm biofilm structure observed when the Δ*araE* biofilm is grown in the presence of L-arabinose.

In the Δ*araE* biofilms grown in the presence of 40 mM L-arabinose, RNA-Seq identified the gene *cyaA* as downregulated -3.2-fold, whose gene product adenylate cyclase generates the second messenger cAMP – the cofactor of cAMP receptor protein (CRP), a global transcriptional regulator that acts as a sensor upon binding intracellular cAMP and affects the expression of other regulatory proteins, including central metabolism (Shimada et al., 2011). Previous studies have shown that cAMP inhibits *csgD* transcription and therefore biofilm formation (Paytubi et al., 2017). As such, we constitutively expressed *cyaA* on a plasmid under the control of the *lacZ* promoter to examine if the downstream production of cAMP would reverse the biofilm phenotype seen when Δ*araE* is grown in the presence of L-arabinose (**Supplemental Figure S3B**). However, not only did constitutive expression of *cyaA* not decrease biofilm formation, it did not circumvent the effect of L-arabinose on the Δ*araE* mutant.

### Biofilm Formation in the Presence of L-Arabinose Correlates to c-di-GMP Production

**Figure 7:**
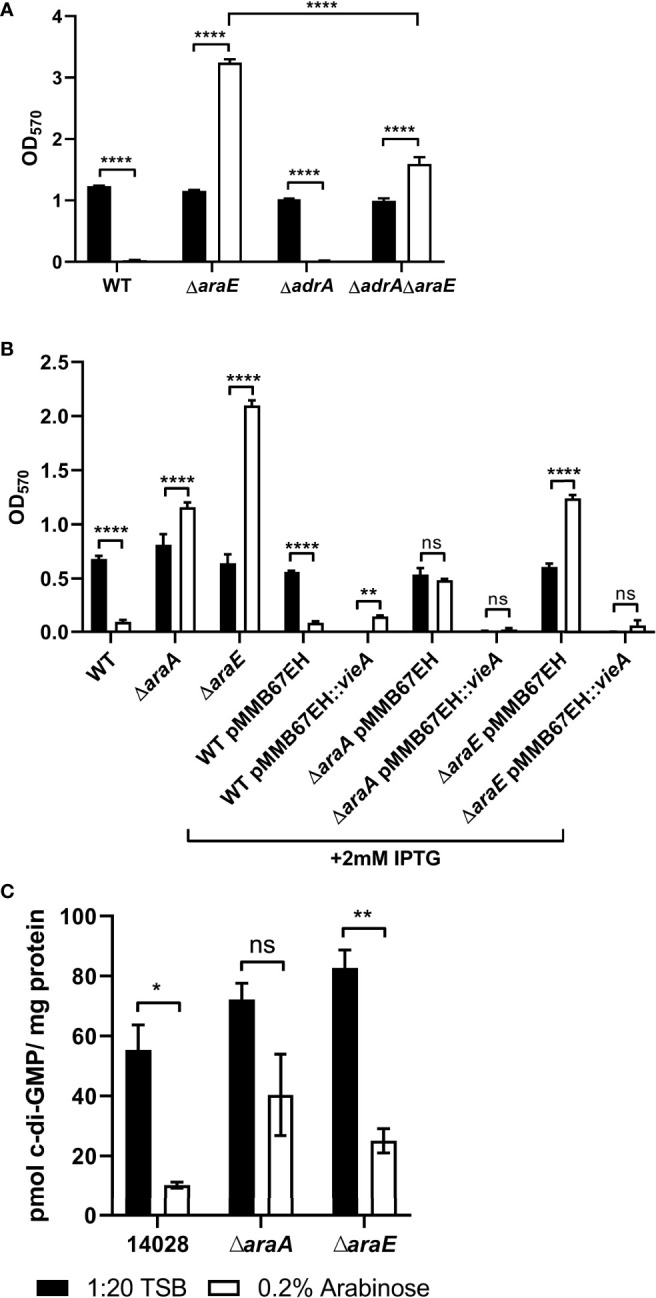
C-di-GMP levels are involved in L-arabinose mediated biofilm formation. WT, Δ*adrA*, Δ*araE* **(A)**, or strains containing *vieA* expressed on a plasmid induced by IPTG **(B)** were grown in 96-well plates in 100 µL 1:20 TSB (black bars) or 1:20 TSB with 0.2% (white bars). After 24 hours, planktonic cells were removed, then biofilms were washed, heat fixed, and stained with crystal violet (CV) for relative biofilm measurement as determined at OD_570_. **(C)** C-di-GMP was extracted from 24 hour biofilms grown in 1:20 TSB (black bars) or 1:20 TSB with 0.2% L-arabinose (white bars) and normalized to total protein concentration. Data are mean ± SD, statistical analyses were done using a two-way ANOVA with Dunnett’s multiple comparisons test NS, not significant (*P* > 0.05); *P < 0.05; **P < 0.01; ****P < 0.0001.

Another gene of interest was *adrA*, which was downregulated -4.3-fold in the WT biofilms grown in 40 mM versus 5 mM L-arabinose. Indirectly induced by CsgD, its gene product is a diguanylate cyclase that produces cyclic-di-GMP (c-di-GMP) and activates cellulose synthesis (Zogaj et al., 2001; Simm et al., 2004). Previous studies have also shown that while the function of CsgD is not dependent on c-di-GMP levels, c-di-GMP does enhance *csgD* expression (Kader et al., 2006; Ahmad et al., 2017), through which c-di-GMP mediates the transition between biofilm formation and virulence (Lamprokostopoulou et al., 2010). No other diguanylate cyclases were upregulated in the RNA-Seq experiment (**Supplemental Table S3**). Therefore, we examined the effect of deleting *adrA* from the WT and Δ*araE* backgrounds in biofilm formation with or without L-arabinose. While Δ*adrA* alone did slightly reduce overall biofilm formation, the absence of AdrA significantly decreased the Δ*araE* biofilm in the presence of L-arabinose, suggesting a role for c-di-GMP in the hyperbiofilm phenotype of the Δ*araE* strain grown with L-arabinose (**Figure 7A**).

Additionally, we used the pMMB67EH plasmid containing the IPTG-inducible *Vibrio cholerae vieA* to further test the role of c-diGMP in biofilm formation. VieA is a two-component response regulator that represses genes involved in *V. cholerae* L-arabinose-mediated biofilm modulation. Its EAL domain functions as a phosphodiesterase and decreases c-di-GMP (Tischler and Camilli, 2004). With this plasmid, addition of IPTG activates *vieA* expression and thus decreases c-di-GMP and typically biofilm formation. When added to the Δ*araE* background, this strain was no longer able to form biofilms in the presence of L-arabinose (**Figure 7B**). This further suggests that c-di-GMP is involved in biofilm formation when grown in the presence of L-arabinose.

To quantify the amount of c-di-GMP present in biofilms with/without L-arabinose and in the Δ*araA* and Δ*araE* mutants, c-di-GMP was extracted from each biofilm and measured using mass spectrometry and normalized to total protein. L-arabinose treatment decreased c-di-GMP levels in WT biofilms. Unexpectedly, c-di-GMP was also reduced in Δ*araE* biofilms grown with L-arabinose (**Figure 7C**). Taken together, the reduction of biofilm formed by WT in the presence of L-arabinose seems to correlate to decreased curli and c-di-GMP but the mechanism by which L-arabinose induces Δ*araE* to form exceptionally large biofilms remains to be determined.

## Discussion

**Figure 8:**
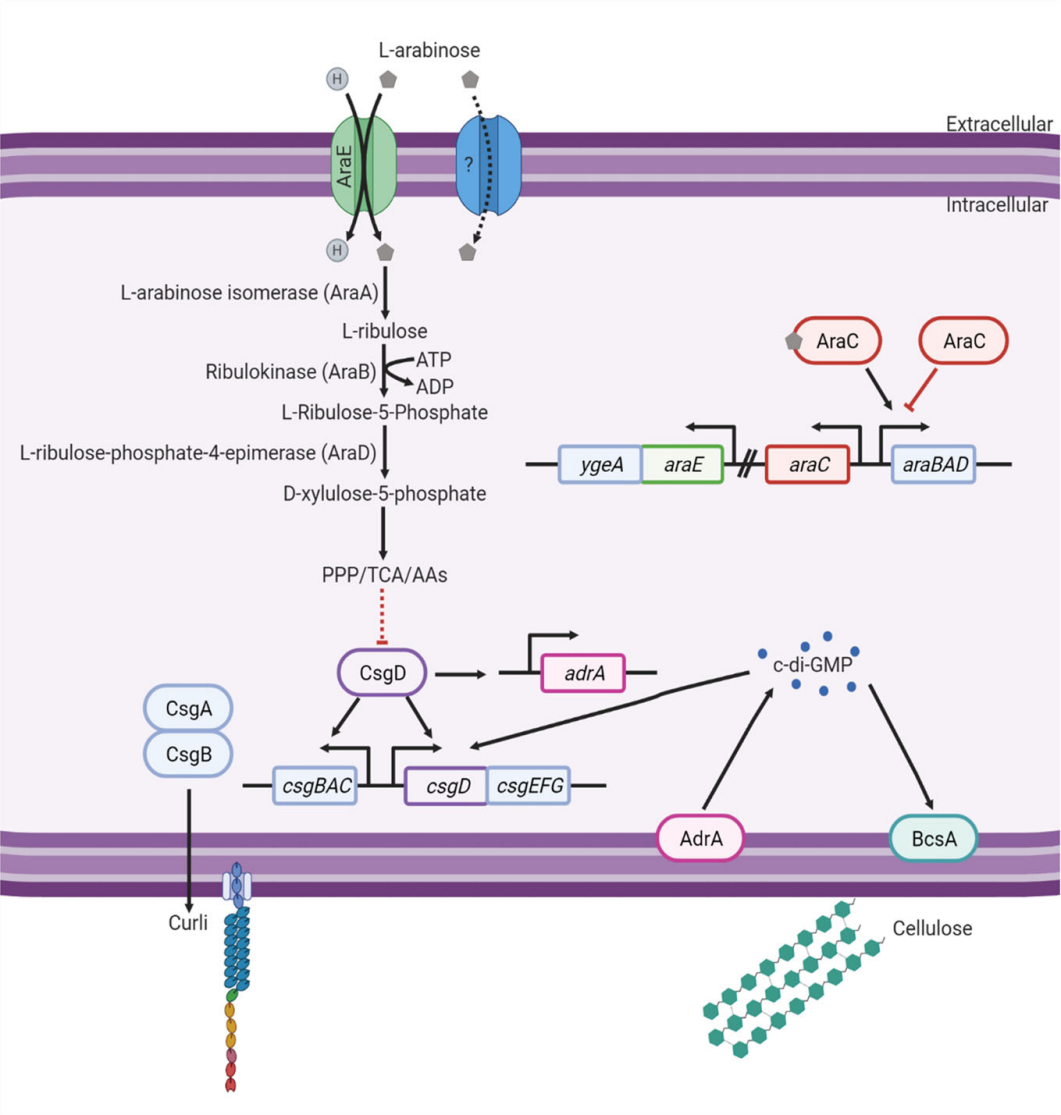
Model of the effect L-arabinose metabolism has on biofilm signaling. Created with BioRender.com.

In this study, we show that L-arabinose metabolism represses biofilm formation but not biofilm dispersal. Additionally, the inability to transport L-arabinose (when not present in high concentrations) *via* AraE results in hyperbiofilm formation. These results may be attributed, in part, to changes in curli gene regulation and intracellular c-di-GMP (**Figure 8**).

In WT, the addition of L-arabinose to biofilm growth media results in decreased biofilm formation, which corresponds to decreased biofilm CFUs and biofilm thickness, but does not affect overall growth or biofilm dispersal. A similar effect was observed when grown in the presence of D-glucose but not D-arabinose. As *Salmonella* cannot metabolize D-arabinose (Gutnick et al., 1969), this suggests that the effect may be related to a sufficient amount of consumable carbon sources present. When grown in such conditions, the cells would not be scavenging for an energy source and may lack the stress factors that drive the transition from planktonic growth into a biofilm. If the ability to metabolize L-arabinose is eliminated by deletion of *araA*, the biofilm forming capabilities and characteristics in the presence of L-arabinose revert to that of WT alone.

Indeed, the metabolism of L-arabinose by WT *S*. Typhimurium negatively affects biofilm formation and promotes planktonic growth, as supported by the downregulation of *csgABC* (curli) and *adrA* (c-di-GMP) observed by RNA-Seq, and the reduction in c-di-GMP levels assessed by mass spectrophotometry. While the function of CsgD is not dependent on c-di-GMP levels, c-di-GMP does enhance *csgD* expression. (Ahmad et al., 2017). Therefore, when c-di-GMP levels are low, *csgD*, encoding a master regulator of biofilm development, is not upregulated.

Further analysis of the RNA-Seq data showed differentially expressed genes similar to those observed in previously published data correlated to poor biofilm formation. This included the upregulation of ATP synthesis (*atpCFG*) (González et al., 2019), asparagine synthesis (*asnB*) (Hamilton et al., 2009), and cysteine biosynthesis (*cysACDHIJPUW*) (Gonzalez-Escobedo, 2013), all of which have been implicated in reducing biofilm formation when induced. In addition, there was an upregulation of genes encoding fimbria proteins (*fimAI*), which is associated with poor biofilm formation (Gonzalez-Escobedo and Gunn, 2013). Meanwhile, ribonucleotide reductase (*nrdEFHI*) were also downregulated in poor biofilms, which is in agreement with previous biofilm studies (Dreux et al., 2015).

Interestingly, the addition of L-arabinose to WT cells also affects host cell invasiveness, but deletion of *prgH* alone (encoding a Type III secretion system [T3SS] structural protein) or the entire SPI-1 operon does not affect biofilm formation. Previous studies have attributed the invasion defect in the presence of L-arabinose to the repression of HilD and thus decreased SPI-1 gene expression and T3SS effector translocation. In agreement, two T3SS proteins (*ssaTU*) previously observed to be downregulated in biofilms (González et al., 2019) were also downregulated in WT in the presence of L-arabinose. Additional studies are needed to determine the mechanism of L-arabinose-mediated repression of *Salmonella* host cell invasion.

Other genes of interest that are upregulated in WT in the presence of L-arabinose by RNA-Seq include glutamate dehydrogenase (*gdhA*) (González et al., 2019), glutathione transport (*yliAB*) (Owens and Hartman, 1986), glycine cleavage (*gcvHPT*) (González et al., 2019), heat shock proteins (*groEL*/*ES*, *htpB*, *ibpA*) (Tang et al., 1997; Tao et al., 2015), hydroxyphenylacetate catabolism (*hpaDEFGHI*) (Prieto et al., 2004) and a PTS operon repressor (*mlc*) (López-Garrido et al., 2015). Downregulated genes of note were involved in proprionate catabolism (*prpBCDE*) (Jia et al., 2017), deoxyribonucleotide metabolism (*nrdEFHI*) (Dreux et al., 2015; Yssel, 2017), sialic acid transport (*nanT*) (González et al., 2019), and a stress induced protein (*ymdF*) (Moshiri et al., 2018). The involvement of these genes in L-arabinose mediated biofilm alteration remains to be examined.

In Δ*araE*, the addition of 0.2% L-arabinose to biofilm growth media results in increased biofilm formation, which corresponds to increased biofilm CFUs, biofilm biomass, and biofilm thickness, but does not affect overall growth or biofilm dispersal. Conversely, the addition of 2% L-arabinose reverts the biofilm forming capabilities and characteristics of Δ*araE* to that of WT in the presence of 0.2% L-arabinose. This suggests that L-arabinose is entering the cell and being metabolized by an alternative pathway, possibly a low affinity transport system (Brown and Hogg, 1972). Future studies to identify such a low affinity transport system could include examining a transposon mutant library in the Δ*araE* background. These double mutants could then be screened for biofilm formation in the presence or absence of both 0.2% and 2% L-arabinose. Candidate mutations would retain an increased biofilm in the presence of both 0.2% and 2% L-arabinose.

Previous studies have shown the importance of the ECM components in biofilm formation, particularly *csgA* (curli), when grown in the presence of 0.1% bile on cholesterol-coated plates (Adcox et al., 2016). Indeed, even the increased biofilm formation seen in Δ*araE* in the presence of L-arabinose decreased when paired with a Δ*csgA* mutant, and is nearly abolished in an Δ*araE* mutant lacking all 4 ECM components (ΔECM). After observing this decrease, it was surprising to also observe a decrease in *csgAC* expression by RNA-Seq analysis of the thicker Δ*araE* biofilm. However, previous studies in our lab observed early activation of the *csgBAC* operon in biofilms grown in M9 supplemented with 10mM D-glucose which then decreased as the biofilm matured after 4 days (González et al., 2019). The RNA-Seq biofilms in this study were grown for 4 days and may have missed potential differential expression during early attachment. Further studies examining expression over time may also show early activation of curli genes and further elucidate the mechanisms involved in biofilm formation in the presence of L-arabinose, particularly in the Δ*araE* background.

Further comparison of the RNA-Seq data showed *cyaA* (cAMP) downregulated in the Δ*araE* strain in the presence of 40mM L-arabinose consistent with previous data (Liu et al., 2020). However, ectopic expression of *cyaA* in Δ*araE* did not alter biofilm formation in the presence of L-arabinose. In addition, *adrA* (c-di-GMP) was shown to be downregulated in WT in media with 40mM L-arabinose (reduced biofilm), but is upregulated in biofilms without L-arabinose (González et al., 2019). We showed that deletion of *adrA* alone did not impact overall biofilm formation, but pairing Δ*adrA* with Δ*araE* resulted in a significant decrease in biofilm formation in the presence of L-arabinose, suggesting involvement of c-di-GMP in the Δ*araE* hyperbiofilm phenotype. The apparent role of c-di-GMP was further implicated upon ectopic expression of the phosphodiesterase *vieA*, which decreased c-di-GMP and abolished not only general biofilm formation, but also the hyperbiofilm phenotype of Δ*araE* in the presence of L-arabinose. Surprisingly, this did not correlate to an observable increase in intracellular c-di-GMP levels in the Δ*araE* strain presence of L-arabinose as measured by mass spectrophotometry. While mass spectrophotometry did demonstrate an expected reduction in c-di-GMP in the WT strain in the presence of L-arabinose, it appears that the hyperbiofilm phenotype of the Δ*araE* strain grown with L-arbainose may be multifactorial. But it would be interesting to examine the levels of c-di-GMP at various timepoints during biofilm formation as it may fluxuate as it matures.

It is also possible that the gene expression changes that impact this phenotype occur early on in biofilm development prior to our RNA-Seq analysis performed after 4 days of growth. However, many of our observed expression changes correlate to biofilm-activated genes in previous studies such formate hydrogenases (*hycCDEFGHI*) (González et al., 2019), glycerol metabolism (*glpABCDEFKQT*) (Chelvam et al., 2015), tryptophan synthesis (*trpA*) (Hamilton et al., 2009), threonate catabolism (*ygbJKLM*) (Chelvam et al., 2015), and a carbon starvation protein (*yjiY*) (Chandra et al., 2017). Previously published downregulated biofilm-related genes also found in our study included pyrimidine salvage (*cdd*, *udp*) (Garavaglia et al., 2012), purine metabolism (*deoAC*) (Yadav et al., 2012), a stress adapter protein (*cpxP*) (Prigent-Combaret et al., 2001), heme biosynthesis (*hemeE*) (Szelestey et al., 2013), SPI-2 type three secretion system (T3SS) proteins (*ssaOU*, *sseI*, *sspH2*) (Desai et al., 2016), and the ascorbate-specific PTS system (*ulaA_2*) (Chelvam et al., 2015; Pandit et al., 2017).

An important factor to also consider is that the RNA-Seq was performed in M9 which differs from the use of 1:20 TSB in most other experiments. While TSB is widely used in bacterial studies, minimal media such as M9 is often used when studying the roles of specific nutrients (Sridhar and Steele-Mortimer, 2016), such as L-arabinose in this study. However, gene expression can change in response to environmental cues, including changes in growth media. Therefore it is possible that despite the phenocopying of arabinose-mediated biofilms in both media conditions, there may be other differences in gene expression between M9 and 1:20 TSB that are unaccounted for.

The mechanism by which the AraE L-arabinose transport mutant forms large biofilms in the presence of L-arabinose remains elusive. When L-arabinose is present at low concentrations but *S*. Typhimurium is unable to transport L-arabinose into the cell *via* AraE, there is potential that an Δ*araE* mutant is responding to carbon starvation by upregulating alternate carbon utilization pathways such as ascorbate and glycerol, despite the lack of presence of these compounds in the M9 growth media. Carbon starvation has been shown to upregulate *yjiY* which is correlated to an increased in *csgD* expression (Chandra et al., 2017) and amino acid catabolism (Hamilton et al., 2009). Additionally, the upregulation of *deoD*, a purine nucleoside phosphorylase (PNP) responsible for scavenging and breaking down nucleotides, would lead to the production of free purine bases and sugars that can be used as a carbon source and has been associated with *Salmonella* biofilm formation (Koopman et al., 2015). But the involvement of these genes in L-arabinose mediated biofilm alteration remains to be examined and further studies are required.

Most importantly, our findings show that the addition of L-arabinose to *S*. Typhimurium biofilm assays alters biofilm formation, suggesting that researchers should use caution when incorporating the L-arabinose inducible pBAD plasmids in biofilm studies. Though not studied here, other genera of bacteria may be similarly affected by L-arabinose. Physiological assays using ectopic expression *via* L-arabinose may also be impacted by other observed metabolic changes. At the very least, necessary controls need to be included in such assays.

## Data Availability Statement

The datasets presented in this study can be found in online repositories. The names of the repository/repositories and accession number(s) can be found in the article/**Supplementary Material**.

## Author Contributions

EV and JG contributed to conception and design of the study. JF and PW analyzed the initial RNA seq data. LO and MP performed the mass spectrometry. EV wrote the first draft of the manuscript. JG, LO, and JF wrote sections of the manuscript. All authors contributed to the article and approved the submitted version.

## Funding

Funded by The National Institutes of Health (R21AI156328; R21AI153752; R01AI116917; R01AI077628 and R01AI143916-01) and Nationwide Children’s Hospital.

## Conflict of Interest

The authors declare that the research was conducted in the absence of any commercial or financial relationships that could be construed as a potential conflict of interest.

## Supplementary Material

Supplementary Figure 1 AraJ and MglC are not involved in L-arabinose low-affinity transport. WT 14028, Δ*araE*, and either **(A)** Δ*araJ*, and Δ*araJaraE* or **(B)** Δ*mglC* and Δ*mglCaraE* were grown in 96-well plates in 100 µL 1:20 TSB (black bars), 1:20 TSB with 0.2% L-arabinose (white bars), or 1:20 TSB with 2% L-arabinose (gray bars). After 24 hours, planktonic cells were removed, then biofilms were washed, heat fixed, and stained with crystal violet (CV) for relative biofilm measurement as determined at OD_570_. Data are mean ± SD, statistical analyses were done using a two-way ANOVA with Dunnett’s multiple comparisons test. Flat bar compares 1:20 TSB to both 0.2% and 2% L-arabinose *****P* < 0.0001.

Supplementary Figure 2 L-arabinose also effects biofilm formation in M9. **(A)** WT 14028, Δ*araA* and Δ*araE* strains were grown in 96-well plates in 100 µL M9 supplemented with varying concentrations of L-arabinose. The concentrations are in mM noted in parentheses. After 24 hours, planktonic cells were removed, then biofilms were washed, heat fixed, and stained with crystal violet (CV) for relative biofilm measurement as determined at OD_570_. **(B)** WT 14028 and Δ*araE* were grown in 96-well plates in 100 µL M9 supplemented with 5 mM or 40 mM L-arabinose. OD_600_ was measured every 30 minutes for 24 hours.

Supplementary Figure 3 YcfR and CyaA are not involved in L-arabinose hyperbiofilm formation. **(A)** WT 14028, Δ*araE*, Δ*ycfR*, and strains containing *ycfR* constitutively expressed on a plasmid were grown in 96-well plates in 100 µL 1:20 TSB (black bars) or 1:20 TSB with 0.2% (white bars). After 24 hours, planktonic cells were removed, then biofilms were washed, heat fixed, and stained with crystal violet (CV) for relative biofilm measurement as determined at OD_570_. **(B)** WT 14028, Δ*araE*, and strain containing *cyaA* constitutively expressed on a plasmid. Data are mean ± SD, statistical analyses were done using a two-way ANOVA with Dunnett’s multiple comparisons test *****P* < 0.0001.
