# Supplementary figures and images for: L-Arabinose Transport and Metabolism in Salmonella Influences Biofilm Formation
Source: Front Cell Infect Microbiol. 2021 Jul 22;11:698146. doi: 10.3389/fcimb.2021.698146 (PMC8341724; doi:10.3389/fcimb.2021.698146)

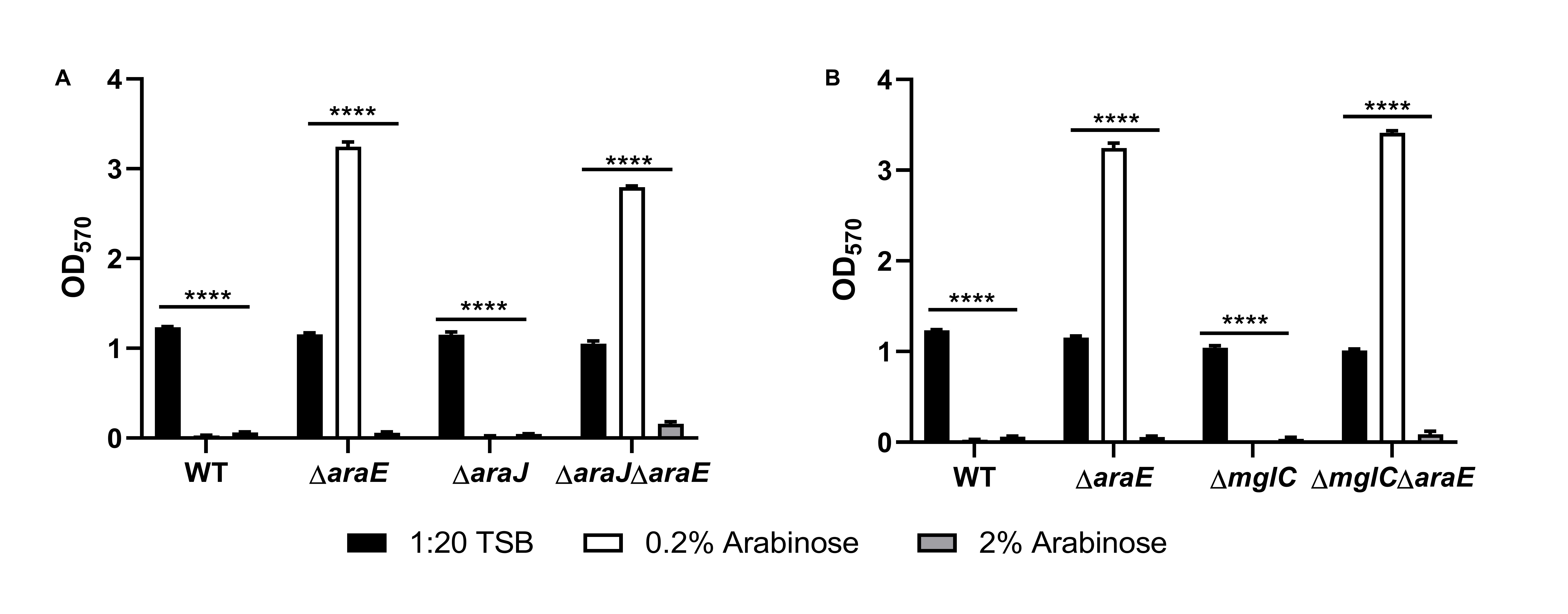

Supplement: Supplementary Figure 1 — AraJ and MglC are not involved in L-arabinose low-affinity transport. WT 14028, ΔaraE, and either (A) ΔaraJ, and ΔaraJaraE or (B) ΔmglC and ΔmglCaraE were grown in 96-well plates in 100 µL 1:20 TSB (black bars), 1:20 TSB with 0.2% L-arabinose (white bars), or 1:20 TSB with 2% L-arabinose (gray bars). After 24 hours, planktonic cells were removed, then biofilms were washed, heat fixed, and stained with crystal violet (CV) for relative biofilm measurement as determined at OD570. Data are mean ± SD, statistical analyses were done using a two-way ANOVA with Dunnett’s multiple comparisons test. Flat bar compares 1:20 TSB to both 0.2% and 2% L-arabinose ****P < 0.0001. [file Image_1.jpeg]

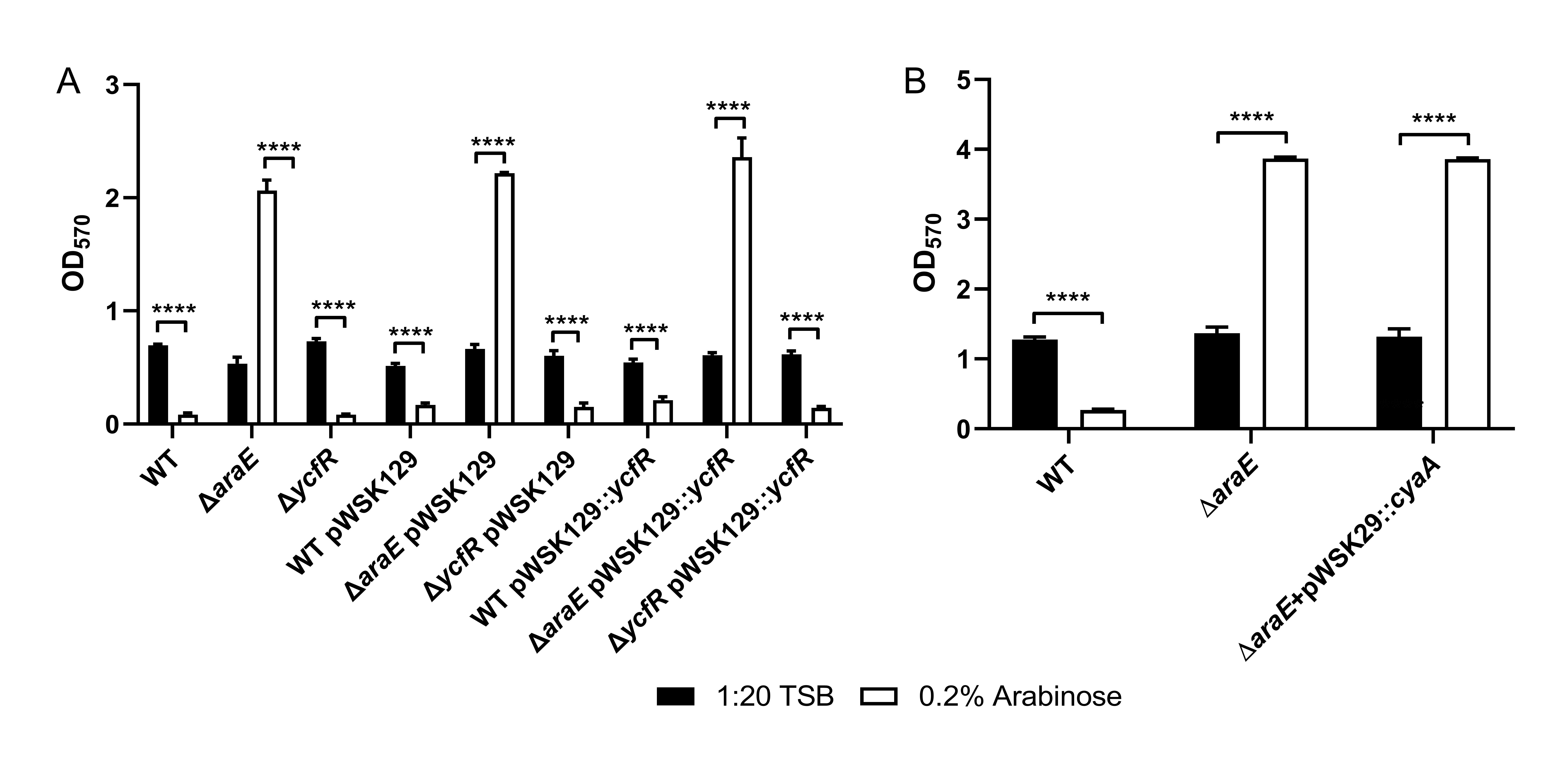

Supplement: Supplementary Figure 3 — YcfR and CyaA are not involved in L-arabinose hyperbiofilm formation. (A) WT 14028, ΔaraE, ΔycfR, and strains containing ycfR constitutively expressed on a plasmid were grown in 96-well plates in 100 µL 1:20 TSB (black bars) or 1:20 TSB with 0.2% (white bars). After 24 hours, planktonic cells were removed, then biofilms were washed, heat fixed, and stained with crystal violet (CV) for relative biofilm measurement as determined at OD570. (B) WT 14028, ΔaraE, and strain containing cyaA constitutively expressed on a plasmid. Data are mean ± SD, statistical analyses were done using a two-way ANOVA with Dunnett’s multiple comparisons test ****P < 0.0001. [file Image_3.jpeg]
