# Supplementary material for: L-Arabinose Transport and Metabolism in Salmonella Influences Biofilm Formation
Source: Front Cell Infect Microbiol. 2021 Jul 22;11:698146. doi: 10.3389/fcimb.2021.698146 (PMC8341724; doi:10.3389/fcimb.2021.698146)
Supplement: Supplementary file 4 [file Table_1.docx]

**TABLE S1**. Strains used in this study^a^

| **Strain** | **Genotype or relevant characteristics** | **Parent/Background** | **Donor** | **Source or Reference** |
| --- | --- | --- | --- | --- |
| JSG047 | DH5α carrying pWSK29 | DH5α |  | (Wang and Kushner 1991) |
| JSG133 | DH5α carrying pWSK129 | DH5α |  | (Wang and Kushner 1991) |
| JSG206 | 14028 *∆phoP* (*phoP*::Tn*10*d::Cam) (CS015) | 14028 |  | (Miller, Kukral, and Mekalanos 1989) |
| JSG208 | 14028 PhoP^C^ (*pho24*) | 14028 |  | (Miller and Mekalanos 1990) |
| JSG210 | *S.* Typhimurium ATCC 14028 | 14028 |  | ATCC |
| JSG1679 | *E. coli* K-12 (BW25141) carrying pKD3::Cam | K-12 |  | (Datsenko and Wanner 2000) |
| JSG1680 | *E. coli* K-12 (BW25141) carrying pKD4::Kan | K-12 |  | (Datsenko and Wanner 2000) |
| JSG1682 | *E. coli* K-12 (BW25113) carrying pKD46 | K-12 |  | (Datsenko and Wanner 2000) |
| JSG1773 | DH5α (BT340) carrying pCP20 | DH5α |  | (Datsenko and Wanner 2000) |
| JSG2963 | 14028 carrying pMMB67EH::*vieA* | 14028 |  | Gift from Rita Tamayo |
| JSG2964 | 14028 carrying pMMB67EH | 14028 |  | Gift from Rita Tamayo |
| JSG3119 | 14028∆*ycfR*::Cam | JSG210 | JSG1679 | (Gonzalez-Escobedo and Gunn 2013) |
| JSG3179 | 14028∆*adrA* | 14028 |  | (Porwollik et al. 2014) |
| JSG3220 | 14028∆*hilA* | 14028 |  | (Porwollik et al. 2014) |
| JSG3377 | 14028*∆prgH*::Kan | 14028 | JSG1680 | (Porwollik et al. 2014) |
| JSG3391 | 14028*∆spiI*::Kan ^a^ | 14028 | JSG1680 | Gift from S. Libby |
| JSG3461 | 14028∆*ycfR* | JSG210 |  | (Gonzalez-Escobedo and Gunn 2013) |
| JSG3529 | 14028 carrying pWSK129 | JSG210 | JSG133 | (Gonzalez-Escobedo and Gunn 2013) |
| JSG3530 | 14028∆*ycfR*::Cam carrying pWSK129 | JSG210 | JSG133 | (Gonzalez-Escobedo and Gunn 2013) |
| JSG3531 | 14028 carrying pWSK129::*ycfR* | JSG210 | JSG3533 | (Gonzalez-Escobedo and Gunn 2013) |
| JSG3532 | 14028∆*ycfR*::Cam carrying pWSK129::*ycfR* | JSG210 | JSG3533 | (Gonzalez-Escobedo and Gunn 2013) |
| JSG3533 | DH5α carrying pWSK129::*ycfR* (pGGE2) | DH5α |  | (Gonzalez-Escobedo and Gunn 2013) |
| JSG3540 | 14028∆*csgA*::Kan | 14028 | JSG1680 | (Porwollik et al. 2014) |
| JSG3672 | 14028∆*yihO* | JSG210 |  | (Marshall and Gunn 2015) |
| JSG3712 | 14028∆*wcaM*::Kan | 14028 | JSG1680 | (Porwollik et al. 2014) |
| JSG3736 | 14028∆*csgA* | JSG3540 |  | (Adcox et al. 2016) |
| JSG3742 | 14028∆*wcaM* | JSG3712 |  | (Adcox et al. 2016) |
| JSG3808 | 14028*∆yihO*::Cam | JSG210 | JSG1679 | (Marshall and Gunn 2015) |
| JSG3834 | 14028 carrying pKD46 | JSG210 | JSG1682 | (Prouty and Gunn 2003) |
| JSG3836 | 14028∆*bcsE*::Cam | JSG210 | JSG1679 | (Adcox et al. 2016) |
| JSG3838 | 14028∆*bcsE* | JSG3836 |  | (Adcox et al. 2016) |
| JSG3841 | 14028∆*wcaM*∆*csgA*∆*yihO*∆*bcsE* | JSG210 |  | (Adcox et al. 2016) |
| JSG3943 | 14028*∆araA*::Cam | JSG210 | JSG1679 | This study |
| JSG3944 | 14028*∆araE*::Cam | JSG210 | JSG1679 | This study |
| JSG3954 | 14028*∆araA* | JSG3943 |  | This study |
| JSG3955 | 14028*∆araE* | JSG3944 |  | This study |
| JSG3958 | 14028*∆araA∆spiI*::Kan | JSG3954 | JSG3391 | This study |
| JSG3959 | 14028*∆araE∆spiI*::Kan | JSG3955 | JSG3391 | This study |
| JSG3960 | 14028*∆araA∆prgH*::Kan | JSG3954 | JSG3377 | This study |
| JSG3961 | 14028*∆araE∆prgH*::Kan | JSG3955 | JSG3377 | This study |
| JSG3965 | 14028*∆prgH*::Kan | JSG210 | JSG3377 | This study |
| JSG3966 | 14028*∆spiI*::Kan | JSG210 | JSG3391 | This study |
| JSG3969 | 14028*∆prgH* | JSG3965 |  | This study |
| JSG3970 | 14028*∆spiI* | JSG3966 |  | This study |
| JSG3973 | 14028*∆araA*∆*csgA*::Kan | JSG3954 | JSG3540 | This study |
| JSG3974 | 14028*∆araE*∆*csgA*::Kan | JSG3955 | JSG3540 | This study |
| JSG3975 | 14028*∆araA*∆*bcsE*::Cam | JSG3954 | JSG3836 | This study |
| JSG3976 | 14028*∆araE*∆*bcsE*::Cam | JSG3955 | JSG3836 | This study |
| JSG4093 | 14028 carrying pFPV25.1 (GFP^C^) | JSG210 | JSG1093 | (González et al. 2019) |
| JSG4244 | 14028*∆araE* carrying pFPV25.1 (GFP^C^) | JSG3955 | JSG1093 | This study |
| JSG4368 | 14028*∆araA*∆*araE* | JSG3954 | JSG3944 | This study |
| JSG4380 | 14028*∆araE* carrying pWSK129 | JSG3955 | JSG133 | This study |
| JSG4381 | 14028*∆araE* carrying pWSK129::*ycfR* | JSG3955 | JSG3533 | This study |
| JSG4414 | 14028*∆araJ*::Cam | 14028 | JSG1679 | (Porwollik et al. 2014) |
| JSG4416 | 14028*∆araJ*::Cam | JSG210 | JSG4414 | This study |
| JSG4418 | 14028*∆araE∆araJ*::Cam | JSG3955 | JSG4416 | This study |
| JSG4431 | 14028*∆mglC*::Cam | 14028 | JSG1679 | (Porwollik et al. 2014) |
| JSG4432 | 14028*∆mglC*::Cam | JSG210 | JSG4431 | This study |
| JSG4434 | 14028*∆araE∆mglC*::Cam | JSG3955 | JSG4431 | This study |
| JSG4436 | 14028∆*wcaM*∆*csgA*∆*yihO*∆*bcsE∆araA*::Cam | JSG3841 | JSG3943 | This study |
| JSG4437 | 14028∆*wcaM*∆*csgA*∆*yihO*∆*bcsE∆araE*::Cam | JSG3841 | JSG3944 | This study |
| JSG4567 | 14028*∆araA∆yihO*::Cam | JSG3954 | JSG3808 | This study |
| JSG4568 | 14028*∆araE∆yihO*::Cam | JSG3955 | JSG3808 | This study |
| JSG4569 | 14028*∆wcaM∆araA*::Cam | JSG3742 | JSG3943 | This study |
| JSG4570 | 14028*∆wcaM∆araE*::Cam | JSG3742 | JSG3944 | This study |
| JSG4595 | 14028*∆araA* carrying pMMB67EH::vieA | JSG3954 | JSG2963 | This study |
| JSG4596 | 14028*∆araE* carrying pMMB67EH | JSG3954 | JSG2964 | This study |
| JSG4599 | 14028*∆araE* carrying pMMB67EH::vieA | JSG3955 | JSG2963 | This study |
| JSG4600 | 14028*∆araE* carrying pMMB67EH | JSG3955 | JSG2964 | This study |
| JSG4603 | 14028*∆adrA∆araE*::Cam | JSG3179 | JSG3955 | This study |
| JSG4699 | DH5α carrying pWSK29::cyaA | DH5α | JSG047 | This study |
| JSG4700 | 14028*∆araE* carrying pWSK29::cyaA | JSG3955 | JSG4699 | This study |
| ^a^ various spontaneous mutations when sequenced | | | | |

REFERENCES

Adcox, Haley E., Erin M. Vasicek, Varun Dwivedi, Ky V. Hoang, Joanne Turner, and John S. Gunn. 2016. “*Salmonella* Extracellular Matrix Components Influence Biofilm Formation and Gallbladder Colonization.” *Infection and Immunity* 84 (11): 3243–51. https://doi.org/10.1128/IAI.00532-16.

Datsenko, Kirill A., and Barry L. Wanner. 2000. “One-Step Inactivation of Chromosomal Genes in *Escherichia Coli* K-12 Using PCR Products.” *Proceedings of the National Academy of Sciences* 97 (12): 6640–45. https://doi.org/10.1073/pnas.120163297.

González, Juan F., Lauren Tucker, James Fitch, Amy Wetzel, Peter White, and John S. Gunn. 2019. “Human Bile-Mediated Regulation of *Salmonella* Curli Fimbriae.” *Journal of Bacteriology* 201 (18). https://doi.org/10.1128/JB.00055-19.

Gonzalez-Escobedo, Geoffrey, and John S. Gunn. 2013. “Identification of *Salmonella Enterica* Serovar Typhimurium Genes Regulated during Biofilm Formation on Cholesterol Gallstone Surfaces.” *Infection and Immunity* 81 (10): 3770–80. https://doi.org/10.1128/IAI.00647-13.

Marshall, Joanna M., and John S. Gunn. 2015. “The O-Antigen Capsule of Salmonella Enterica Serovar Typhimurium Facilitates Serum Resistance and Surface Expression of FliC.” *Infection and Immunity* 83 (10): 3946–59. https://doi.org/10.1128/IAI.00634-15.

Miller, S. I., A. M. Kukral, and J. J. Mekalanos. 1989. “A Two-Component Regulatory System (PhoP PhoQ) Controls *Salmonella* Typhimurium Virulence.” *Proceedings of the National Academy of Sciences of the United States of America* 86 (13): 5054–58. https://doi.org/10.1073/pnas.86.13.5054.

Miller, S. I., and J. J. Mekalanos. 1990. “Constitutive Expression of the PhoP Regulon Attenuates Salmonella Virulence and Survival within Macrophages.” *Journal of Bacteriology* 172 (5): 2485–90. https://doi.org/10.1128/jb.172.5.2485-2490.1990.

Porwollik, Steffen, Carlos A. Santiviago, Pui Cheng, Fred Long, Prerak Desai, Jennifer Fredlund, Shabarinath Srikumar, et al. 2014. “Defined Single-Gene and Multi-Gene Deletion Mutant Collections in Salmonella Enterica Sv Typhimurium.” *PloS One* 9 (7): e99820. https://doi.org/10.1371/journal.pone.0099820.

Prouty, A. M., and J. S. Gunn. 2003. “Comparative Analysis of Salmonella Enterica Serovar Typhimurium Biofilm Formation on Gallstones and on Glass.” *Infection and Immunity* 71 (12): 7154–58. https://doi.org/10.1128/iai.71.12.7154-7158.2003.

Wang, R. F., and S. R. Kushner. 1991. “Construction of Versatile Low-Copy-Number Vectors for Cloning, Sequencing and Gene Expression in Escherichia Coli.” *Gene* 100 (April): 195–99.
