# Supplementary material for: L-Arabinose Transport and Metabolism in Salmonella Influences Biofilm Formation
Source: Front Cell Infect Microbiol. 2021 Jul 22;11:698146. doi: 10.3389/fcimb.2021.698146 (PMC8341724; doi:10.3389/fcimb.2021.698146)
Supplement: Supplementary file 5 [file Table_2_v1.docx]

**TABLE S2**. Primers used in this study^ab^

| **Primer** | **Sequence** |
| --- | --- |
| JG2801 – araA-F deletion | 5’-GCAATCCTGACTCATTAAGGACACGACA**ATG**ACGATTTTTGTGTAGGCTGGAGCTGCTTC-3’ |
| JG2802 – araA-R deletion | 5’-AGTGCCACCGGGCAATCCGTTTCACCAA**TTA**ACGTTTGAACATATGAATATCCTCCTTAG-3’ |
| JG2803 – araA-F outer | 5’-CAGGCGCAACGCTTCGAACAGCTTTACCGCCGCTACCAGC-3’ |
| JG2804 – araA-R outer | 5’-CCCTGCCGGATGGCGCTTCGCTTATCCGGCCTACGAGCG-3’ |
| JG2805 – araE-F deletion | 5’-ATGTCTTACTCTGTTGTGGCAGGAAAAT**ATG**GTCTCTATTGTGTAGGCTGGAGCTGCTTC-3’ |
| JG2806 – araE-R deletion | 5’-CAGGCGTCATCCGGCATGGGAGGGGGGA**TTA**CACGCCAATCATATGAATATCCTCCTTAG-3’ |
| JG2807 – araE-F outer | 5’-CCATCACATATCGCTATAGCGTAGCCATTTAATCC-3’ |
| JG2808 – araE-R outer | 5’-CCCAGCTCATCCCCCCCAACAGTCCGATCGTTTTCACTGC-3’ |
| JG3100 – mglC-F deletion | 5’-TTTGCACCTTTAAGATCAGGGGCTCCTT**ATG**AGTGCGTTAGTGTAGGCTGGAGCTGCTTC-3’ |
| JG3101 – mglC-R deletion | 5’-TGATAAGCAAGGCAATAGGTCTGGATAA**CTA**CTTCTTACGCATATGAATATCCTCCTTAG-3’ |
| JG3102 – araJ-F outer | 5’-GAATACACAAAGACGGATAAGGAATGGTATTACCTCGCGC-3’ |
| JG3103 – araJ-R outer | 5’-GCTGATGAAATCGTTTGTTTTTGGCGGCGAGTGCGAGACG-3’ |
| JG3104 – araJ-F deletion | 5’-ACATGCGCCTTTATTTTCGGCAGGTGGT**ATG**AAAAAAGTTGTGTAGGCTGGAGCTGCTTC-3’ |
| JG3105 – araJ-R deletion | 5’-GGCGTAACGTTGTTAGCCGGATGGCGCG**TCA**TCCGGCAACCATATGAATATCCTCCTTAG-3’ |
| JG3106 – mglC-F outer | 5’-CTGTTAGGGATTACCGATCGTATCCTGGTGATGAGTAACG-3’ |
| JG3107 – mglC-R outer | 5’-GGTCATGTCTGCCCGGTCGCCTGTATTGATTTAGGCGAGG-3’ |
| JG3198 – adrA-F outer | 5’-TTCTAGAATTTGGGAAAATTGTTTCTAAATGTTCCCAAAA-3’ |
| JG3199 – adrA-R outer | 5’-TCAGTAAATCCTCGAGCCCGGCTGGACGTCATGCCGCCAC-3’ |
| JG3200 – cyaA-F outer | 5’-GACGTCTAGAAACATCAGGCGATACGTCTTGTACCTCTAT-3’ |
| JG3201 – cyaA-R outer | 5’-GGCCGGGCAGCTCGAGCCGGCGCAGCACTTACGAAAAATA-3’ |
| ^a^Start or stop codons are in boldface font  ^b^Cassette sequences are underlined | |
